# Very high MHC Class IIB diversity without spatial differentiation in the mediterranean population of greater Flamingos

**DOI:** 10.1186/s12862-017-0905-3

**Published:** 2017-02-20

**Authors:** Mark A. F. Gillingham, Arnaud Béchet, Alexandre Courtiol, Manuel Rendón-Martos, Juan A. Amat, Boudjéma Samraoui, Ortaç Onmuş, Simone Sommer, Frank Cézilly

**Affiliations:** 1 University of Ulm, Institute of Evolutionary Ecology and Conservation Genomics, Albert-Einstein Allee 11, D-89069 Ulm, Germany; 2 Université de Bourgogne, Equipe Ecologie Evolutive, UMR CNRS 6282 Biogéosciences, 6 bd. Gabriel, 21000 Dijon, France; 3 Centre de Recherche de la Tour du Valat, Le Sambuc, 13200 Arles, France; 4 Leibniz Institute for Zoo and Wildlife Research, Evolutionary Genetics, Alfred-Kowalke-Str. 17, D-10315 Berlin, Germany; 5 Berlin Center for Genomics in Biodiversity Research (BeGenDiv), D-14195 Berlin, Germany; 6 R.N. Laguna de Fuente de Piedra, Consejería de Medio Ambiente y Ordenación del Territorio, Junta de Andalucía, Apartado 1, E-29520 Fuente de Piedra, (Málaga), Spain; 7 Department of Wetland Ecology, Estación Biológica de Doñana, (EBD-CSIC), calle Américo Vespucio s/n, E-41092 Sevilla, Spain; 8 Natural History Museum, Faculty of Sciences, Department of Biology, Ege University, Bornova, İzmir, Turkey; 9 Center of Excellence for Research in Biodiversity, King Saud University, Riyadh, Saudi Arabia; 10 Laboratoire de recherche et de conservation des zones humides, University of Guelma, Guelma, Algeria; 11 Institut Universitaire de France, Paris, France

**Keywords:** MHC genes, Allelic diversity, Pathogen-mediated balancing selection, Local adaptation, Greater flamingos

## Abstract

**Background:**

Selective pressure from pathogens is thought to shape the allelic diversity of major histocompatibility complex (MHC) genes in vertebrates. In particular, both local adaptation to pathogens and gene flow are thought to explain a large part of the intraspecific variation observed in MHC allelic diversity. To date, however, evidence that adaptation to locally prevalent pathogens maintains MHC variation is limited to species with limited dispersal and, hence, reduced gene flow. On the one hand high gene flow can disrupt local adaptation in species with high dispersal rates, on the other hand such species are much more likely to experience spatial variation in pathogen pressure, suggesting that there may be intense pathogen mediated selection pressure operating across breeding sites in panmictic species. Such pathogen mediated selection pressure operating across breeding sites should therefore be sufficient to maintain high MHC diversity in high dispersing species in the absence of local adaptation mechanisms. We used the Greater Flamingo, *Phoenicopterus roseus*, a long-lived colonial bird showing a homogeneous genetic structure of neutral markers at the scale of the Mediterranean region, to test the prediction that higher MHC allelic diversity with no population structure should occur in large panmictic populations of long-distance dispersing birds than in other resident species.

**Results:**

We assessed the level of allelic diversity at the MHC Class IIB exon 2 from 116 individuals born in four different breeding colonies of Greater Flamingo in the Mediterranean region. We found one of the highest allelic diversity (109 alleles, 2 loci) of any non-passerine avian species investigated so far relative to the number of individuals and loci genotyped. There was no evidence of population structure between the four major Mediterranean breeding colonies.

**Conclusion:**

Our results suggest that local adaptation at MHC Class IIB in Greater Flamingos is constrained by high gene flow and high MHC diversity appears to be maintained by population wide pathogen-mediated selection rather than local pathogen-mediated selection. Further understanding of how pathogens vary across space and time will be crucial to further elucidate the mechanisms maintaining MHC diversity in species with large panmictic populations and high dispersal rates.

**Electronic supplementary material:**

The online version of this article (doi:10.1186/s12862-017-0905-3) contains supplementary material, which is available to authorized users.

## Background

Genetic diversity is an essential component of the adaptive potential of populations [1, 2]. The major histocompatibility (MHC) genes code for glycoproteins which present antigens to T-cells playing an integral role in the adaptive immune response [3]. The MHC genes form a multigene family, and are known to be among the most polymorphic genes of the vertebrate genome, with most populations exhibiting a high level of MHC allelic diversity in terms of both allele number and sequence divergence [4, 5]. This multigene family has therefore become an important model of adaptive variation in natural populations of vertebrates [4, 5].

The maintenance of MHC polymorphism is generally attributed to powerful pathogen-mediated balancing selection, with gene conversion and recombination, maternal-foetal interactions and sexual selection also thought to play a role [4, 5]. Since selection from local pathogens may be sufficiently strong to change MHC allele frequencies within the short evolutionary time of a few generations [6–8], we can expect local pathogen-mediated selection at the MHC to be stronger than either neutral forces (such as gene flow and genetic drift) or pathogen-mediated selection at the scale of the metapopulation. Such local pathogen-mediated selection can occur if there is spatial and temporal variation in pathogen-mediated selection (“fluctuating selection”) [9] and/or local cyclical selection between host and pathogen (“negative frequency-dependent selection”) [10–12], and both mechanisms are thought to play a crucial role in maintaining MHC diversity. Indeed, stronger population structure at the MHC than at neutral markers have been demonstrated in numerous species that show some level of population structure at neutral markers (reviewed in [5]). However, the occurrence of local adaptation at the MHC in species with high gene flow remains to be investigated.

Long-lived and dispersing species with large geographical distributions, often associated with panmixia at neutral markers, face a particular challenge: individuals are likely to encounter during their life heterogeneous environmental conditions, each associated with distinct pathogen communities [13]. Indeed, species that are migratory [14, 15] and have large geographical distributions [14, 16, 17] are known to have more diverse parasitic communities. As a consequence of encountering more diverse parasitic communities, migratory or long-distance dispersing species may be under more intense pathogen mediated selection than resident species. In support of the latter, a recent comparative study has found that the rate of non-synonymous nucleotide substitution across the antigen binding regions of the MHC class IIB (a signal of balancing selection) is significantly higher in migratory species [18]. The latter study also found that colonial species had a higher rate of non-synonymous nucleotide substitution, which suggests that the high contact between conspecifics in colonial species leads to elevated transmission rates and therefore increased balancing selection of MHC genes [18]. However, the high gene flow of high dispersing species is likely to disrupt local adaptation and other pathogen-mediated balancing selection mechanisms are likely to be maintaining MHC diversity. For instance, frequency-dependent selection may be operating across breeding sites in panmictic species. Another non-mutually exclusive hypothesis, which could also be operating across breeding sites in panmictic species, is the “overdominant heterozygote advantage” hypothesis. The latter proposes that MHC heterozygotes will be fitter than the fittest MHC homozygote because they will be able to recognize a greater diversity of parasites [19, 20]. Higher MHC allelic diversity can thus be expected in colonial and long-distance dispersing species with little or no spatial differentiation between breeding sites than locally adapted resident species, based on the idea that higher pathogen-mediated balancing selection is operating across breeding sites in panmictic species. In addition, such long-range dispersing species should have no MHC population structure since gene flow prevents local adaptation.

**Fig. 1:**
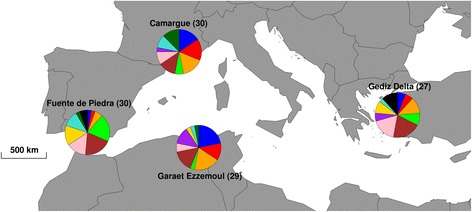
Pies representing MHC Class IIB exon 2 allele frequencies for alleles with a frequency of at least 10% within the four studied breeding colonies of the Greater Flamingos across the Mediterranean basin (alleles with a frequency below 10% are not represented). Each colour represents a different allele

To investigate MHC diversity and population structure in a long-lived colonial species with high gene flow, we used data from Gillingham et al. [21], which genotyped MHC Class IIB exon 2 of 116 Greater Flamingos across four breeding colonies spread across the entire range of the species in the Mediterranean basin (Fig. 1). Non-random patterns of natal and breeding dispersal between breeding colonies has been shown by capture-mark-recapture studies [22], which may favour local divergent adaptations [23]. However, molecular data on 13 microsatellite loci and two mitochondrial markers were consistent with strong gene flow and a panmictic genetic structure of the population at the scale of the Mediterranean basin [24].

## Methods

### Samples

Samples were collected between 1998 and 2009, at four breeding colonies across the Mediterranean basin breeding range (Table 1; Fig. 1): Garaet Ezzemoul, northern Algeria (35°53’N, 06°30’E); Camargue, southern France (43°25’N, 04°38’E); Fuente de Piedra, southern Spain (37°06’N, 04°45’W); and Gediz Delta, western Turkey (38°32’N, 26°52’E).

**Table 1:** Summary statistics of MHC Class IIB exon 2 variation for the four breeding colonies of Greater Flamingos

Sampling site	Year of sampling	*N*	*m*	π (±SD)	*S*	Mean # alleles per individual	Mean AA_dist_ (±SE)	Mean AA_distSelect_ (±SE)	Private alleles	APD (±SE)
Algeria	2008 & 2009	29	56	0.107 (±0.003)	64	3.34 (±0.15)	0.237 (±0.005)	0.641 (±0.016)	8	94.33 (±2.56)
France	1998	30	63	0.107 (±0.002)	65	3.47 (±0.11)	0.236 (±0.004)	0.633 (±0.013)	12	95.52 (±2.32)
Spain	2007	30	55	0.104 (±0.003)	66	3.43 (±0.11)	0.228 (±0.004)	0.637 (±0.012)	8	94.25 (±2.53)
Turkey	2007 & 2009	27	56	0.105 (±0.002)	65	3.52 (±0.13)	0.228 (±0.006)	0.647 (±0.019)	11	93.53 (±2.68)
Total	1998–2009	116	109	0.105 (±0.001)	68	3.44 (±0.06)	0.233 (±0.002)	0.639 (±0.007)	39	94.50 (±1.24)

*N*: number of individuals. *m*: number of alleles. π (±SD): population nucleotide diversity and standard deviation. AA_dist_ (±SE): mean amino acid divergence within individual and standard error of the mean. AA_distSelect_ (±SE): mean amino acid divergence within individual for sites under positive selection according to Gillingham et al. [21] and standard error of the mean. APD (±SE): average per cent difference and standard error of the mean

### MHC Class IIB exon2 genotyping

Details of MHC Class IIB exon 2 genotyping can be found in Gillingham et al. [21] which genotyped the individuals used in this study using barcoded PCRs in 454 Titanium pyrosequencing run. Briefly, 163–165 bp of the 267–270 bp of the MHC Class IIB exon 2 was amplified which code for nine positively selected codon sites [21]. Assignment of barcoded sequences to individual barcodes and allele validation was carried out with the aid of SEquence Sorter & AMplicon Explorer (SESAME) [25]. We used a slightly modified version of Sommer et al.’s [26] pipeline and allele validation followed the following rules: 1) all reads whose length suggested a shift in reading frame during sequencing were discarded; 2) all variants that were represented only once in the run (i.e. singletons) were assumed to be artefacts; 3) we assumed that artefactual variants should occur at lower frequencies than true variants and we used the conservative *T*_*2*_ threshold recommended by Galan et al. [27] with *F*_*ij*_ of 4% or above to assign reads as alleles; 4) all sequences that had a 1–2 base pair difference to one of the validated alleles within the sample were assumed to be artefacts; 5) all sequences that had more than a 2 base pair difference from validated alleles and were also validated as alleles if they were classified as alleles in other individuals; 6) the remaining sequences that had more than a 2 base pair difference from a validated allele were validated as alleles only if their frequency exceeded any artefact; 7) we assumed that chimeras were always present together with the true alleles from which they were formed and in lower frequency. Accordingly chimeras were easily identified when reads were aligned within individuals in SESAME and were discarded if in lower frequency than source validated alleles. Gillingham et al. [21] reported a maximum of 4 alleles per individual suggesting two loci for this marker [21]. Of the 126 individuals genotyped, we used in subsequent analyses the 116 for which the authors achieved a 99.9% confidence in allele calling (>300 reads per individual) (see Gillingham et al. [21] for details of allele calling and simulations). Based on 10 duplicates and 2 triplicates Gillingham et al. [21] found a repeatability of 100% in allele calling.

### Population differentiation analyses

First, we estimated nucleotide diversity and number of segregating sites using DnaSP version 5 [28]. Second, we measured MHC population differentiation. There are many measures of population differentiation and ways of partitioning genetic variance, with constant debate and research among population geneticists about which measure is the best to use in which circumstance [29–33]. Multilocus genes such as the MHC add the additional complication that their complex nature often violates assumptions required to apply several of these methods (e.g. Hardy-Weinberg equilibrium, linkage disequilibrium, mixed inheritance patterns). A further complication arises when alleles cannot be assigned to loci, as is often the case for MHC datasets in non-model organisms, preventing the identification of heterozygote and homozygote genotypes. As a result, allele frequencies cannot be accurately estimated as the frequency of common alleles is underestimated and that of rare alleles overestimated. We therefore chose to rely on the metric *Rho* (also known as *p* statistic) devised by Ronfort et al. [34] for genotype data with multiple alleles per locus in each individual as found in polyploidy species. The *Rho* statistic is independent of both the ploidy level and the amount of within-individual diversity. This statistic has also recently been demonstrated by simulation to be less biased when assumptions of diploid inheritance are violated than Nei’s G_ST_ [35], G″_ST_ [36] and Jost’s *D* [29, 32]. We estimated the *Rho* statistic in conjunction with traditional *Fst* [37] to measure MHC population differentiation using the SPAGeDi software [38]. We carried out this analysis on individual MHC Class IIB exon 2 alleles based on the full length nucleotide sequence, allelic lineages based on codon sites which were shown to be under positive selection by Gillingham et al. [21] (i.e. different sequences were considered the same allele if they did not differ in positively selected codon sites) and allelic lineages based on the full length translated amino acid sequences.

When using markers that are highly diverse such as the MHC, many alleles may only be sampled once, even when sample sizes are fairly large, and, in extreme cases, analyses based solely on allele frequencies might be largely ineffective [39, 40]. Therefore, we also used two other methods to detect genetic differentiation between populations. The first is Hudson’s [39] nearest-neighbour statistic (*S*_*nn*_) which measures how often the “nearest neighbours” of sequences (i.e. the most similar sequence(s) of each sequence) are within the same geographical locality. Sequence similarity is determined as the number of base differences per sequence. For a dataset of sequences collected from *n* individuals (including identical sequences from different individuals which are the same allele), *S*_*nn*_ is estimated as:

$$ {S}_{n n} = {\displaystyle \sum_{j=1}^n}{X}_j/ n $$

where, *X* is the number of nearest neighbour belonging to the same geographical locality divided by the total number of nearest neighbour for sequence *j*. Hence, if *X*_*j*_ is equal to 1, then all nearest sequences belonged to individuals sampled within the same geographical locality; and, conversely if *X*_*j*_ is equal to 0, all nearest sequences were from a different geographical locality. In a pairwise comparison between populations, a *S*_*nn*_ that is significantly greater than 0.5 therefore indicates evidence of some population structure, as does a global *S*_*nn*_ which is significantly greater than 0.25 when simultaneously comparing four populations. The *S*_*nn*_ statistic was estimated using the PopGenome package [41] in R and significant departure from predicted values given panmixia were estimated by permutation using 1000 reshuffling in R [42]. We carried out this analysis on both full sequences and on sequences of codon sites which were shown to be under positive selection by Gillingham et al. [21].

The second statistic that we used which does not solely rely on allele frequency was the average percentage difference (APD) which has previously been used in studies investigating MHC population structure [43, 44]. We calculated this statistic as described by Yuhki & O’Brien [43]. Specifically, APD estimates the average percentage in the number of alleles not shared between two individuals within a population as:

$$ A P{D}_k = {\displaystyle \sum_{i=1}^N}{\left({D}_{a b}/\left({A}_a+{A}_b\right)\right)}_i/ N $$

where for population *k*, *N* is the number of pairwise comparisons, *D*_*ab*_ is the number of alleles not shared between individual *a* and individual *b*, *A*_*a*_ and *A*_*b*_ are the total number of unique alleles in individuals *a* and *b*. APD therefore measures within-population genetic variation. We tested whether between breeding colonies APD was significantly different from within breeding colonies APD by permutation using 100,000 reshuffling. APD calculations were performed using R [42].

Finally, we estimated MHC heterozygosity with three metrics that did not require the assignment of MHC alleles to loci (since this is not possible in our dataset [21]): (i) the number of MHC alleles per individual; (ii) the mean amino acid allele divergence within an individual over the entire translated sequences (of the 163–165 bp exon 2 nucleotide sequences; AA_dist_), and (iii) the mean amino acid allele divergence within an individual over sites found to be under positive selection by Gillingham et al. [21] (AA_distSelect_). We obtained the proportion of base pair differences between sequences required to compute AA_dist_ and AA_distSelect_ using MEGA 7 [45]. We tested differences in the number of MHC alleles per individual between breeding colonies using a generalized linear model (GLM) with a Poisson distribution and a log link. For testing differences in AA_dist_ and AA_distSelect_ between breeding colonies we used a GLM model with a binomial distribution (since AA_dist_ and AA_distSelect_ are averages of proportional data) and a logit link function. All GLMs were fitted in R [42].

### Microsatellite genotyping and heterozygosity correlation with MHC Class IIB exon 2

The 116 samples used for Greater Flamingo MHC Class IIB exon 2 differentiation in this study were previously selected for microsatellite genotyping by Geraci et al.[24] for 13 loci: PrA2, PrD3, PrD4, PrD5, PrD7, PrD9, PrA102, PrA110, PrA113, PrC109, PrD108, PrD121 and PrD126. Nine individuals were excluded from this analysis because fewer than 8 microsatellite loci were successfully genotyped for these individuals. In order to compare MHC differentiation to differentiation at neutral markers we estimated *F*_*ST*_ [37] for microsatellite data differentiation as calculated by the SPAGeDi software [38].

### Comparison with other avian non-passerine species of MHC Class IIB exon 2 allelic diversity

In order to compare allelic diversity of greater flamingos with other non-passerine species we searched the literature of other species for which at least 100 individuals were genotyped. We recorded the number of individuals genotyped, the number of loci found (estimated as the number of alleles per individual) and the number of alleles reported. We also calculated the proportion of alleles per individual and the proportion of alleles per individual and loci. We excluded passerines from our study for the following reasons: (1) the evolution and structure of passerine MHC is completely different from other avian species [46], with passerines having a much higher number of copy number variation [46–50] suggesting that macroevolutionary patterns (i.e. gene duplication history) may lead to very different signals of balancing selection between passerines and other birds [49] and (2) passerines have a large number of pseudogenes which is likely to bias true functional MHC allele diversity and render hazardous a comparison in allelic diversity between passerines and other birds, who tend to have only 2–3 functional loci (Table 2). For example the collared flycatcher, *Ficedula albicollis*, appears to have at least 9 functional MHC class IIB loci but also appears to have at least 15 MHC class IIB loci that are pseudogenes [47]. However since alleles cannot be assigned to loci in almost all MHC passerine studies, alleles that appear functional (no frame shift in sequences or premature stop codon) may in fact be in non-functional loci and *vice versa* [49].

**Table 2:** Number of alleles for MHC Class IIB exon2 relative to the number of loci and individuals genotyped in non-passerine birds reported in the literature with 100 or more individuals genotyped, showing species name (common name and Latin name), number of individuals genotyped in the study, maximum number of loci based on the maximum number alleles found within an individual in the population, number of alleles found in the study, the ratio between number of alleles and number of individuals, the ratio between number of alleles, number of individuals and number of loci, whether the species is migratory and/or long distance disperser and the citation of the study

Species (Common name)	Species (Latin name)	# of ind.	# of loci	# of alleles	# of alleles /# of ind.	# of alleles /# of ind./# of loci	Migratory/long-distance disperser	Study
Greater Flamingo	*Phoenicopterus roseus*	116	2	109	0.940	0.470	Yes	This study
Lesser Kestrel	*Falco naumanni*	121	1	103	0.851	0.851	Yes	Alcaide et al., 2008 [55]
Magellanic Penguin	*Spheniscus magellanicus*	100	1	45	0.450	0.450	Yes	Knafler et al., 2012 [77]
Eurasian Coot	*Fulica atra*	906	3	265	0.292	0.097	Partially^a^	Alcaide et al., 2014 [54]
Great Snipe	*Gallinago media*	175	2	50	0.286	0.143	Yes	Ekblom et al., 2007 [78]
Greater Prairie Chicken	*Tympanuchus cupido*	182	2	30	0.165	0.082	No	Minias et al., 2016 [79]
Leach's Storm Petrel	*Oceanodroma leucorhoa*	188	2	24	0.128	0.064	Yes	Dearborn et al., 2016 [80]
Chinese Egret	*Egretta eulophotes*	172	5	20	0.116	0.023	Yes	Lei et al., 2016 [81]
Grey Partridge	*Perdix perdix*	108	2	12	0.111	0.056	No	Promerová et al., 2013 [82]
Blakiston’s Fish Owl	*Bubo blakistoni*	174	8	19	0.109	0.014	No	Kohyama et al., 2015 [83]
Red Grouse	*Lagopus lagopus*	296	3	28	0.095	0.032	No	Meyer-Lucht et al., 2016 [84]
Attwater’s Prairie Chicken	*Tympanuchus cupido*	142	2	5	0.035	0.018	No	Bateson et al., 2016 [85]

^a^Not migratory in the studied population but makes nomadic dispersal movements according to changing water levels and seasonal rainfall. Also fully migratory in other parts of the species distribution

## Results

As reported in Gillingham et al. [21], we detected a very high diversity of MHC Class IIB exon 2 with 109 alleles at two loci among 116 individuals (Table 2). The mean number of alleles per individual was 3.44 (Table 1), and 63 individuals had 4 alleles (54%), 42 individuals had 3 (36%), 10 individuals had 2 (9%), and 1 individual had 1 (1%). The number of alleles identified per population was between 55 and 63 identified alleles (Table 1). Out of 109 alleles identified from nucleotide sequences across the four breeding sites, 39 were private alleles (Table 1), however all of these alleles were rare and only present within either a single individual (34 alleles) or two individuals (5 alleles). The 109 alleles from nucleotides sequences translate into 104 different amino acid sequences. When comparing with other non-passerine avian species, allelic diversity was higher than all species that had been sampled more than greater flamingos (*n* > 116) except for the Eurasian coot (Table 2). When rarefying coot sample size to 116 (resampling 10000 times with no replacement) median number of alleles was 130 (95% confidence intervals was 119–142). When rarefying the flamingo dataset (resampling 10000 times with no replacement) to the sample of the two species in Table 2 with a smaller dataset, greater flamingo allelic diversity remained much greater than those species (*n* = 100, median number of alleles = 104, 95% confidence intervals = 100–107; *n* = 108, median number of alleles = 107, 95% confidence intervals = 104–109).

There was either little or no differentiation (slightly negative values equivalent to zero) between breeding colonies regardless of whether *Fst* or *Rho* was investigated (Table 3a and b) and global *Rho* and *Fst* values were very small and not significantly different from 0 (20,000 permutations: *Rho* = 0.0001, *p* = 0.929; *Fst* = 0.0005, *p* = 0.570). Similar results were found when investigating alleles called from sequences of sites under positive selection (Table 3b; global *Rho* = 0.0007, *p* = 0.827; global *Fst* = 0.0010, *p* = 0.540) and when investigating sequences from amino acid sequences (see Additional file 1: Table S1; global *Rho* = 0.0005, *p* = 0.834; global *Fst* = 0.0007, *p* = 0.528).

**Table 3:** Pairwise *Rho* statistic [34] of MHC Class IIB Exon 2 for alleles called from full nucleotide sequences (below diagonal) and alleles called from nucleotide sequences of sites under positive section (above diagonal) (a); pairwise *F*_*ST*_ statistic [37] of MHC Class IIB Exon 2 for alleles called from full nucleotide sequences (below diagonal) and alleles called from nucleotide sequences of sites under positive section (above diagonal) (b); pairwise *S*_*nn*_ statistic [39] of MHC Class IIB Exon 2 for full length nucleotide sequences (below diagonal) and for nucleotide sequences of sites under positive section (above diagonal) (b) and, pairwise *F*_*ST*_ values for 13 microsatellite loci (above diagonal) for four breeding colonies of Greater Flamingos across the Mediterranean basin

	Algeria	France	Spain	Turkey
a. *Rho* statistic for MHC Class IIB exon 2				
Algeria		−0.0018	0.0137	−0.0003
France	−0.0049		0.0002	−0.0016
Spain	0.0135	0.0038		−0.0075
Turkey	−0.0018	−0.0017	−0.0096	
b. *F*_*ST*_ statistic for MHC Class IIB exon 2				
Algeria		−0.0012	0.0043	0.0006
France	−0.0020		−0.0001	0.0008
Spain	0.0045	0.0010		−0.0007
Turkey	0.0006	0.0007	−0.0014	
c. *S*_*nn*_ statistic for MHC Class IIB exon 2				
Algeria		0.494	0.524	0.480
France	0.494		0.503	0.504
Spain	0.533	0.489		0.489
Turkey	0.515	0.498	0.475	
d*. F*_*ST*_ statistic for 13 microsatellite locus				
Algeria				
France	0.0062			
Spain	0.0042	0.0063		
Turkey	−0.0027	−0.0020	−0.0020	

Note that all the *Rho* and *F*_*ST*_ statistic values are close to zero (negative values are equivalent to 0). None of the differentiation values were significantly different from 0 regardless of the statistic (*Rho* or *F*_*ST*_) or the marker investigated (MHC Class IIB exon 2 or microsatellite markers). Similarly none of the *S*_*nn*_ statistic values were significantly different from 0.5, the predicted value of panmixia between two populations

Further supporting no population structure at the MHC Class IIB exon 2, global *S*_*nn*_ did not significantly deviate from the predicted value for a panmictic population of 0.25 when analysing full length sequences (*S*_*nn*_ = 0.239; *p* = 0.714) and sequences known to be under positive selection (*S*_*nn*_ = 0.246; *p* = 0.608). Similarly pairwise comparison between breeding colonies never significantly deviated from the predicted panmictic value of 0.5 (Table 3c). Average percentage difference (APD) was very high suggesting very high within-population MHC variation (Table 1). Furthermore, APD within colony was not significantly different from APD between breeding colonies (*p* = 0.590) and a similar result was found when analysing APD from amino acid sequences (*p* = 0.493). The mean number of alleles per individual (*χ* ^2^ = 0.132; df = 3; *p* = 0.988), mean AA_dist_ (*χ* ^2^ = 3.069; df = 3; *p* = 0.381) and mean AA_distSelect_ (*χ* ^2^ = 0.449; df = 3; *p* = 0.930) were not significantly different between breeding colonies.

## Discussion

To the best of our knowledge, the MHC allelic diversity in greater flamingos is one of the highest reported so far (this study & [21]) relative to the number of individuals genotyped in non-passerine birds, despite the fact that they appear to harbour only two MHC Class IIB loci (Table 2). Only Coots have slightly higher MHC allelic diversity than Greater flamingos, with 130 alleles (95% confidence intervals was 119–142) compared to the Flamingo’s 109 when rarefied to the same sample size, although Coots harbour three loci. Furthermore, the fact that allele calling is based on 163–165 bp of the 267–270 bp exon 2 sequence suggests that we may be slightly underestimating allelic diversity since alleles may differ in regions outside the amplified segment. The variation we found in the number of alleles per individual can be explained by either variation in loci copy number as reported in certain bird species e.g. [51, 52] or balancing selection maintaining identical alleles across duplicated loci e.g. [53]. Alternatively, we cannot exclude that our design of primers was not optimal and may have led to some allele dropout. Indeed designing optimal primers without any allele dropout is notoriously difficult to achieve in non-model organisms [26]. However the latter is unlikely to bias population differentiation analyses since the same primers were used across all individuals and populations.

Passerines frequently appear to also present a very high diversity of MHC alleles [46–50] but this generally seems to be due to their high number of MHC gene copies [49]. Furthermore due to the high number of pseudogenes reported in passerines, unbiased functional allelic diversity of these species is unclear and difficult to quantify. Two other examples of non-passerine bird species with a high allelic MHC diversity are the Eurasian Coot, *Fulica atra*, [54] (265 alleles; 906 individuals; 3 loci) and the Lesser Kestrel *Falco naumanni* [55] (103 alleles; 121 individuals; 1 loci). Like Greater Flamingos, the Eurasian Coot has a large geographical distribution and form large populations [54], whilst the colonial Lesser Kestrel is a long distance migratory species and forms small populations [55]. One possibility is that high gene flow across breeding sites in the Mediterranean population of Greater Flamingos is relatively recent and that range-wide MHC diversity arose through local adaptation. However, we believe the latter to be highly unlikely for the following reason. Geraci et al. [24] have shown that flamingo populations have undergone a bottleneck followed by rapid growth and expansion, with the average time since expansion being estimated to be 696 421 yr (90% CI: 526 316–1 131 579 yr), suggesting that high gene flow in this species is not recent. A possible increase in gene flow remains possible since over the last three centuries wetlands and favourable nesting places for flamingos have become scarce [56]. However, there is no reason to believe that the species was less nomadic in the ancient past than today. Our study therefore supports that in Greater Flamingos, although high dispersal rates disrupts the local adaptation mechanisms that are predicted to maintain MHC diversity, pathogen-mediated balancing selection at the scale of the large and panmictic Mediterranean population is sufficiently strong to generate high MHC allelic diversity. However, a greater characterisation of MHC diversity of migratory and non-migratory bird species than is currently available in the literature, followed by a meta-analysis, would be required to formally test whether ecology and life history traits of species (such as migration, long distance dispersal, wide geographic distribution and colonial breeding) predicts population wide MHC allelic diversity.

Theory predicts that local adaptation and divergent selection between populations should be favoured when gene flow is at an intermediate level [23]. Such intermediate levels of gene flow may occur between breeding sites of colonial breeding species if philopatric behaviour is coupled with regular dispersal [57]. In lesser kestrels, pathogen-mediated balancing selection at the local scale of the population appears to be maintaining high allelic diversity (stronger local population structure at MHC markers than at neutral makers) [55]. A similar result was reported in the migratory but philopatric loggerhead sea turtles (*Caretta caretta*) which showed distinct MHC differentiation between breeding colonies, despite close proximity between sites (80–260 km) [58]. However, evidence that local adaptation can occur despite high gene flow has only been reported in a few studies of marine fish [59–62]. It is also worth noting that we could find no evidence of temporal variation in MHC allele frequency. The Greater Flamingo colony in France was sampled 9–11 years prior to other breeding colonies in our study. The slow reproductive rate of flamingos means that this represents approximately a difference of a single generation with other breeding colonies [22]. Indeed, Pradel et al. [63] demonstrated that at age 10, virtually all individuals had attempted to breed at least once and about 90% of them have already done so at age 9. Thus our results tentatively suggests that allele frequency does not significantly shift during this short time period as reported in species with faster reproductive rates [8]. However more generations from the same geographical locality are needed to confirm the latter. Nonetheless, in the case of Greater Flamingos in the Mediterranean basin, given the lack of spatial MHC population structure, high allelic MHC diversity appears to be maintained by overdominant heterozygote advantage and/or population wide frequency dependent selection rather than local fluctuating selection and/or local variation in frequency dependent selection.

The lack of locally adapted alleles may be explained by gene swamping, whereby specific local polymorphism is lost because gene flow is larger than selection [64]. Furthermore, for local population structure at the MHC to occur there needs to be strong spatial variation in parasite communities [5]. While Greater Flamingos occupy a large diversity of wetland habitats across the Mediterranean, the parasite communities that flamingos encounter remain by and large unknown. However, a strong driver of MHC class IIB are likely to be the multiple species of helminths that Greater Flamingos are known to be parasitized by [65], with artemia species consumed by Greater Flamingos known to be the intermediate host [66]. Indeed parasitic helminths are known to cause significant pathology in birds which can result in lower fat content [67], increased chick mortality [68] and lower reproductive success [69]. Furthermore, migratory species are known to harbour a larger diversity of nematodes [14, 15], in particular species with wide geographic distributions and using multiple aquatic habitats [14] such as is the case for Greater Flamingos. It is therefore possible that flamingos do encounter a strong variation of parasites at different sites in the Mediterranean, but significant breeding dispersal [22, 70] leads to sufficiently strong gene flow to disrupt any possible local adaptation. In this case, dispersal may also enable Greater Flamingos to escape from locally prevalent pathogens that are highly adapted to the hosts.

Alternatively, the high dispersal rates of flamingos may act to facilitate the dispersal of parasites themselves, and, hence, homogenise parasite communities across the Mediterranean. Furthermore it has been suggested that saline wetlands may harbour fewer parasites than freshwater wetlands [71]. For example, Mendes et al. [72] found that species of wading birds inhabiting saline habitats had fewer blood parasite infections than species from freshwater habitats. Indeed, despite the screening of chicks, studies have never detected blood parasites in Greater Flamingos in the Mediterranean [56, 73]. Regarding helminths, a comparative study by Poulin [74] using data from 389 parasitic species of cestodes, trematodes and nematodes in 158 bird species found that a positive correlation between heavy infections in local host populations and the ability to exploit many host species. The latter is suggestive that helminth infections that affect most fitness may be widespread rather than locally constrained. Therefore, the diversity and heterogeneity of parasites encountered by flamingos may be comparatively low and, in turn, the cost of dispersing, in terms of encountering new parasites that they are not immune to, may also be comparatively lower than for inland species. Indeed, the high dispersal rates encountered in Greater Flamingos are on par with the ones found in marine colonial birds that tend to have a higher dispersal rates than other migratory avian species (reviewed in [75]). In the absence of local variation in parasite communities, the benefits of dispersing, such as finding alternative breeding and feeding sites to avoid high competition at the natal site (especially for young inexperienced birds), may outweigh the costs.

## Conclusion

Regardless of whether parasites vary across time and space in flamingos, the very high diversity of MHC Class IIB alleles observed in this study suggests strong pathogen-mediated balancing selection at the scale of the whole population. Given the lack of local MHC population structure, MHC diversity appears to be maintained by overdominant heterozygote advantage and/or population wide frequency dependent selection rather than local fluctuating selection and/or local variation in frequency dependent selection. Further understanding of how pathogens vary across space and time will be crucial to further elucidate the mechanisms maintaining MHC diversity in large panmictic populations with high dispersal rates [5].

## Additional file

Additional file 1: Table S1. Pairwise *Rho* statistic [34] (a) and pairwise *F*_*ST*_ statistic [37] of MHC Class IIB Exon 2 for alleles called from amino acid sequences for four breeding colonies of Greater Flamingos across the Mediterranean basin. (DOCX 18 kb)
