# Supplementary material for: Very high MHC Class IIB diversity without spatial differentiation in the mediterranean population of greater Flamingos
Source: BMC Evol Biol. 2017 Feb 20;17:56. doi: 10.1186/s12862-017-0905-3 (PMC5319168; doi:10.1186/s12862-017-0905-3)
Supplement: Additional file 1: Table S1. — Pairwise Rho statistic [34] (a) and pairwise F ST statistic [37] of MHC Class IIB Exon 2 for alleles called from amino acid sequences for four breeding colonies of Greater Flamingos across the Mediterranean basin. (DOCX 18 kb) [file 12862_2017_905_MOESM1_ESM.docx]

Table S1 : Pairwise *Rho* statistic [34] (a) and pairwise *F_ST_* statistic [37] of MHC Class IIB Exon 2 for alleles called from amino acid sequences for four breeding colonies of Greater Flamingos across the Mediterranean basin.

|  | Algeria | France | Spain |
| --- | --- | --- | --- |
| 1. *Rho* statistic for MHC Class IIB exon 2 | | | |
| France | -0.0047 |  |  |
| Spain | 0.0133 | 0.0038 |  |
| Turkey | -0.0018 | -0.0014 | -0.0073 |
| 1. *F_ST_* statistic for MHC Class IIB exon 2 | | | |
| France | -0.002 |  |  |
| Spain | 0.0044 | 0.001 |  |
| Turkey | 0.0004 | 0.0008 | -0.0008 |

Note that all the *Rho* and *F_ST_* statistic values are close to zero (negative values are equivalent to 0). None of the differentiation values were significantly different from 0 regardless of the statistic (*Rho* or *F_ST_*).
